# Silicon as a Vegetable Crops Modulator—A Review

**DOI:** 10.3390/plants8060148

**Published:** 2019-05-31

**Authors:** Prashant Kaushik, Dinesh Kumar Saini

**Affiliations:** 1 Instituto de Conservación y Mejora de la Agrodiversidad Valenciana, Universitat Politècnica de València, 46022 Valencia, Spain; 2 Department of Plant Breeding and Genetics, Punjab Agricultural University, Ludhiana 141004, India; dineshsaini-pbg@pau.edu

**Keywords:** vegetable, silicon, biotic, abiotic, stress

## Abstract

Vegetables require an optimum supply of mineral elements like silicon (Si). Si is second to oxygen in its abundance in the earth crust, and its role is quite significant in tackling biotic and abiotic stresses of vegetables. Si application also improves several agronomic and quality traits of vegetables. Hence, Si application is recommended as a strategy for the improvement of vegetable crops production. Although the research about the role of Si in vegetable dicots still lags far behind than cereals. Recently, omics-based approaches were used to provide a deeper understanding of the role of Si in vegetable protection. Here, we have compiled the studies focusing on the role of Si for vegetables, thus, enabling all of the important information regarding the effect Si application to vegetables at one place.

## 1. Introduction

Vegetables are vulnerable to a wide range of biotic and abiotic stresses; to overcome these stresses, vegetables requires an optimum supply of macro and micronutrients [1,2,3]. Silicon (Si) is crucial for plants; moreover, Si is always present in large quantities near the plant roots. [4]. Si occurs as silicates or silicon oxides, and around 27.7 percent of earth’s crust is composed of Si, but still, the available forms of Si meant for plants are scarce. Plants commonly use monosilicic acid (H_4_SiO_4_) as the source of Si and H_4_SiO_4_ exists in the liquid form in soil. Moreover, the concentration of H4SiO4 is not correlated to the total Si quantity of the soil. [5,6]. Regardless of the plentiful publications that establish Si application advantages in agriculture, Si is not regarded as an essential element. Si is classified as a quasi-essential element for plants [7]. Therefore, based on Si uptake, vegetables are divided into three groups, i.e., active, passive, and rejective. Whereas, based on the accumulation of Si in the cell wall apoplast, vegetables are classified as accumulators, excluders, and intermediate types [8,9]. The role of Si on plant health has been tested under open field conditions, hydroponic cultures, and under greenhouse/glasshouse environment [10]. Still, presently there are a limited number of studies which demonstrate there are advantages of Si application for greenhouse crops.

Meeting the growing demand for vegetables under situations of biotic and abiotic stresses is a big challenge. Si application is considered as an eco-friendly approach for crop production; therefore, Si application is commonly recommended under package and practices for cereals. Likewise, in vegetables, Si application has been documented to reduce the attack of diseases [11]. For example, potassium silicate treatment of pea seedlings was observed to increase chitinase and β-1,3-glucanase activity against the fungal pathogen *Mycosphaerella pinodes* and it is the causes of blight disease in pea [12]. Similarly, Si application has considerably reduced the root rot and powdery mildew disease in cucumber and the rust disease of cowpea [13,14,15]. Moreover, nano-silicon application can prevent postharvest diseases of vegetables [16,17]. In this direction, studies have also demonstrated that higher Si content in plant tissues reduced the incidence of several insect pests. [18]. Correa et al. [19] reported that soil or as a foliar spray of Si as calcium silicate to cucumber plants increases the mortality of the nymphs of *Bemisia tabaci*. 

Correspondingly, several abiotic stresses affecting the vegetables are eradicated by the application of Si [20]. Si application was reported to alleviate high-temperature stress in vegetables [21]. Si application protects vegetables against the UV-B radiation by increasing photosynthesis and antioxidant levels [22]. Recently, omics-based approaches were applied to gain a genomic level perception of the mechanisms by which Si application aids vegetables in unfavorable circumstances. [23]. Heavy metals are detrimental for plant growth, and plants commonly accumulate heavy metals that are hazardous to human health. Si application is useful in reducing heavy metal toxicity. However, the role of Si for vegetables (dicots) is not well studied as compared to the model plants like Arabidopsis and rice. Subsequently, in many of the recent reviews on the role of Si in plants, the vegetable crops are underrepresented [24,25,26]. Therefore, we have structured our review focusing on the role of Si application for vegetables, especially, in tackling biotic and abiotic stresses, as well as the role of Si application on agronomic and quality traits of vegetables, thus, collating all of the important information regarding the effect of Si application to vegetables in one place. 

## 2. Biotic Stress 

### 2.1. Fungal Pathogens

#### 2.1.1. Cucurbitaceae

The efficacy of Si application has been reported against many fungal pathogens. Using scanning electron microscopy, Samuels et al. [27] observed an overall negative correlation between the amount of Si and the growth of the causal agent of powdery mildew disease of cucumber (*Sphaerotheca fuliginea*). El-Samman [28] reported controlling the root rot of cucumber caused by *Pythium aphanidermatum* and *Fusarium solani* using the soluble formulation of Si. Foliar and root applied Si was determined to control the powdery mildew disease of cucumber [14]. Further, increased activities of plant protectants like superoxide dismutase (SOD), catalase (CAT), peroxidase (POD) in addition to the contents of ascorbate (AsA) and glutathione (GSH) were observed in the leaves of cucumber with Si application [29]. Si application was also effective against the oxidative stress induced by *Phytophthora melonis* infection in cucumber [30]. 

In 2006, Heine et al. [31] determined the ability of symplastic Si to reduce the spread of *P. aphanidermatum* in the roots of tomato and bitter gourd [32]. In 2010, Yu et al. [33] reported that Si application enhanced cucumber seedling growth and resistance level against *Fusarium oxysporum* f.sp. *cucumerinum* [34]. Si application as sodium silicate was found to be more effective against powdery mildew in melon as compared to nano-sized Si [35]. The effect of Si application on the severity and incidence of powdery mildew and quality traits like total soluble solids and dry matter contents have been reported for melon landraces carosello (3.6 °Brix and 4.81 g 100 g^−1^ FW) and barattiere (4.0 °Brix and 4.95 g 100 g^−1^ FW) [36,37]. 

Increased activities of biochemical defense enzymes viz., peroxidase, polyphenol oxidase, and pathogenesis-related proteins (chitinase and β-1,3-glucanase) have been observed in bitter gourd with the application of soluble Si [38]. Guo et al. [39] used Si (silicon oxide and sodium silicate) for the control of postharvest pink rot (*Trichothecium roseum*) in Chinese cantaloupe. Si nutrient solution, have been tested for enhancing the tolerance to powdery mildew of hydroponically grown zucchini squash (*Cucurbita pepo* L.) [40]. The effectiveness of soil amendments for providing Si nutrition has been tested on pumpkin in contrast to the powdery mildew [41].

#### 2.1.2. Solanaceae 

In tomato, Diogo et al. [42] devised an alternative strategy for the management of fusarium crown and root rot (*Fusarium oxysporum* f.sp. *radices lycopersici*) using Si application to the tomato plant. [43]. Moreover, a positive effect of Si supplementation on post-harvest quality traits has been observed in tomato [44]. 

In capsicum and chilli pepper, the potential of Si application to decrease the symptoms of Phytophthora blight (*Phytophthora capsici*) development has also been confirmed [45]. Jayawardana et al. [46] reported Si induced resistance against anthracnose disease (*Colletotrichum gloeosporioides*) in chilli pepper. 

#### 2.1.3. Leguminosae 

In soybean, absorption of Si in leaves of different soybean cultivars was quantified and correlated with the ability to enhance the resistance against soybean (*Glycine max*) rust (*Phakopsora pachyrhizi*) [47,48]. Similarly, a study indicated that the delay in disease onset was the possible cause of the final reduction in area under soybean rust progression curve [49].

### 2.2. Bacterial Pathogens

Si application is also effective against bacterial pathogens. For the first time, Dannon and Wydra [50] reported a significant effect of Si application against bacterial wilt disease of tomato (*Ralstonia solanacearum*). In this direction, Wydra et al. [51] found that Si accumulation mainly happened in the roots, and a negative correlation was reported between root Si content and bacterial growth. Ghareeb et al. [52] reported the up-regulated expression of the jasmonic acid/ethylene marker genes (*JERF3*, *TSRF1*, and *ACCO*) with Si application that induced resistance in tomato plants against *R. solanacearum* infestation [53]. With Si application, a significant boost in activities of enzymes viz., soil urease and soil acid phosphatase were reported under pathogen-inoculated conditions [54]. The resistance of tomato leaves to bacterial wilt mediated by Si application has been associated with the activation of defense-related enzymes such as peroxidase (POD) and phenylalanine ammonia lyase (PAL) [55].

### 2.3. Insect Pest and Nematodes

Studies have shown that Si application can increase the degree of resistance of host plants to insect pests. In this direction, the effect of Si application for resistance, against the whitefly (*Bemisia tabaci*) has been evaluated in tomato and cucumber [19,56,57]. Si application diminished the whitefly population on cucumber plants by reducing the insect oviposition, increasing growth cycle, and by causing high mortality at the nymph stages [19]. Whereas in soybean, Si application did not affect insect oviposition preferences but caused significant mortality at the nymph stages [56]. Recently, Callis-Duehl et al. [57] studied the role of Si application against *D. balteata* and *B. tabaci* of cucumber. Plant protection against insect pests with Si application is further correlated with the amount of increment of biochemical compounds like indols [58]. Recently, Dugui-Es et al. [59] demonstrated the effect of Si concentration and the frequency of application in managing the root-knot nematode, *Meloidogyne incognita*, in cucumber. The observed effects of Si application on biotic stresses faced by vegetables are presented in Table 1.

## 3. Abiotic Stresses

### 3.1. Salinity 

Salinity is a significant cause of yield losses in vegetables [76,77]. Salt stress results in the cations build-up that causes toxicity to the plant roots [78,79,80,81]. Salinity drastically affects the vegetables fresh and dry weight, photosynthetic rate, mesophyll conductance, and photosynthetic water use efficiency [82]. Several studies have reported the effect of Si application on salinity stress in vegetable crops (Table 1). Si mediated alleviation of salinity stress is associated with, a significant increase in the activities of antioxidants and decrease in the contents of electrolytic leakage percentage [83]. Likewise, the increase in activities of antioxidants like superoxide dismutase (SOD), catalase (CAT), was reported in spinach and bitter gourd under salinity [84,85]. The relationship between the amounts of antioxidants and plant growth under salinity stress has been studied for cucumber [86,87] and soybean [88].

Si application improved leaf turgor potential (42%), net photosynthesis rates (20%), water use efficiency (17%) and the ratio between plant dry matter and plant water uptake (16%) in tomato [89]. It has also been concluded that exogenous application of Si in combination with phyto-extracts of *Melia azadirachta* (Chinaberry) can effectively alleviate salinity-induced hazardous effects in pea [90]. Tantawy et al. [91] demonstrated that nano-Si is more effective and efficient in mitigating salinity stress in sweet pepper plants. Similarly, the use of nano-SiO_2_ has been reported in squash for activating the defense mechanisms of plants against salinity [92]. 

The mechanism of Si mediated salt tolerance is still not fully understood, and the possible role of Si in alleviating salt-induced osmotic stress with the underlying mechanism is still unexplored. Although, based on a study conducted on cucumber plants, it was suggested that Si application improved the salt tolerance by enhancing root water uptake, and also by up-regulating of aquaporin gene expression [93]. Si application during salinity stress prevented oxidative damage by increasing the activities of antioxidant enzymes and recovered the nutrient imbalance in *C. annuum* [94]. Si application increased the accumulation of polyamine in cucumber plants for salt tolerance [95].

### 3.2. Drought 

Adequate regulation of plant nutrients may be helpful to maintain or even improve the plant water status thereby making the plant tolerant to drought stress. Si has been reported to confer tolerance to drought by regulating the leaf relative water content, transpiration, and stomatal conductance of plants [96,97]. Shen et al. [88] observed significant effects of Si application on photosynthesis and antioxidant parameters (viz., catalase, peroxidase) of soybean seedlings grown under drought stress. Si application mediated alleviation of drought stress on growth has been confirmed in soybean [98]. 

Likewise, application of exogenous Si improved seed germination and alleviated oxidative stress at the seedling stage of tomato [99] and by increasing the net photosynthetic rate in tomato leaves under water stress [100,101]. Shi et al. [102] suggested the role of Si-mediated decrease in membrane oxidative damage in increasing the root hydraulic conductance and water uptake hence improving water stress tolerance in tomato plants. Recently in 2017, Cao et al. [103] showed the role of changes in radial hydraulic conductivity and cell wall stability with Si application in tomato. 

### 3.3. Other stresses

For osmotic stress studies, effects of Si on photosynthesis of young cucumber seedlings [104], and the activity of antioxidant enzymes in cucumber seedlings have been evaluated [105,106]. Whereas, for chilling stress, Liu et al. [107] showed that exogenous Si leads to greater deposition of endogenous Si and thereby increases antioxidants; and reduces the lipid peroxidation induced by chilling in cucumber.

### 3.4. Mineral Toxicity

#### 3.4.1. Aluminum

Si has been used in vegetable crops for alleviating the toxic effect of aluminum. For the first time in soybean, Baylis et al. [108] showed the alleviation of Al toxicity by Si. On these lines, Bityutskii et al. [109] highlighted the importance of both Fe and Si supply in exclusion of Al under acidic conditions from cucumber plants. Recently, Dorneles et al. [110] demonstrated that Si partially alleviated the damage caused by Al in the root growth parameters in potato via the elevated activity of antioxidant enzymes such as SOD and POD.

#### 3.4.2. Manganese

In the case of manganese (Mn) toxicity, a study showed that Si supply alleviated Mn toxicity by the detoxification of apoplastic Mn [111]. In various studies, this alleviation of Mn toxicity by Si supply is related to the significant increase in the activities of antioxidants such as PPO, PODs, etc. [112,113,114,115]. In 2016, Dragišić Maksimović et al. [116] observed an enhanced cell wall stability owing to inert deposition of Si in the leaf cell walls of cucumber resulting in the decreased amount of toxic free Mn within the plant tissues. 

#### 3.4.3. Cadmium

The high amount of cadmium (Cd) is hazardous for vegetables, and Si has been used to ameliorate its effects in plants [117]. In cucumber, application of Si under cadmium stress protected the photosynthetic machinery from damages and improved the activities of nitrogen metabolism enzymes such as nitrogen reductase (NR) and glutamine synthetase (GS) [118]. Likewise, Wu et al. [119] also confirmed that Si application was reducing Cd uptake by roots in cucumber; whereas, in tomato, Si application was decreasing root-to-shoot Cd transport.

#### 3.4.4. Ammonium

**Figure 1:**
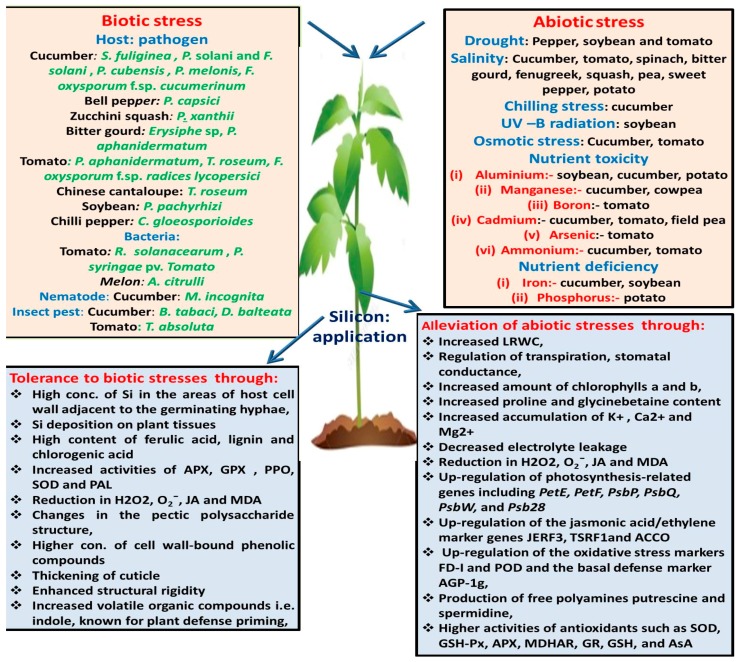
Schematic representation of various biotic and abiotic stresses overcome by Si application, along with the changes that take place after Si application.

Excessive ammonium is associated with various physiological disorders in plants. Role of Si application has been investigated to minimize these disturbances in cucumber and tomato. Campos et al. [120] reported that the application of Si, independent of the cucumber variety, mitigates the toxicity of ammonium and thereby enhances the dry matter of cucumber plants. Barreto et al. [121] recommended the use of Si in the nutrient solution (Si = 1mmolL^−1^) for the tomato plants grown under ammonium stress. The observed effects of Si application on abiotic stresses faced by vegetables are presented in Table 2 and Figure 1.

## 4. Methods of Silicon Application on Vegetable Crops

Various methods have been employed for applying the Si on plants such as Si solution, Si fertilizers, and foliar spray. The foliar spray could be an efficient method of application of Si, but it has not been adequately tested. In this direction, an effort was made by Wolff et al. [64] who evaluated the efficiency of foliar applications of two commercially available Si-based products viz. Carbon Silpower® and Carbon Defense for their effect in reducing powdery mildew development in commercial greenhouse cucumber production. Results showed the starring role of foliar spray of Si for significantly reducing the severity and incidence of disease.

Si is commonly applied in the form of a solution. To our knowledge, for the first time Samuels et al. [27] raised cucumber plants in media supplemented with 100 ppm SiO2, (+Si) and studied the distribution pattern of Si in cucumber leaves during infection under powdery mildew fungus. Whereas foliar sprays with Si compounds are also applied. Further foliar sprays can be classified mainly into four categories, such as (a) foliar sprays with silicates *viz.* calcium silicate on melon [72], (b) foliar sprays with silicic acid viz. spray on soybean plants [56], (c) foliar sprays with other Si compounds, such as silica nanoparticles (nano-SiO2) on cucumber plants [1]; (d) foliar spray of commercially available Si-based products such as Carbon Silpower® and Carbon Defense® [64]. Nowadays, researchers are more focused on the use of nano form of Si for alleviating the effects of salt stress.

## 5. Omics-Based Studies

Some omics-based studies have been conducted in various vegetable crops to identify the differentially expressed genes to study the effect of Si application. For example, in the case of tomato, Kurabachew et al. [70] performed a transcriptome analysis in tomato plants treated with Si following inoculation with *R. solanacearum* and reported 174 differentially regulated genes (113 up-regulated and 61 down-regulated). Functional characterization of genes showed that most of the up-regulated genes were involved in defense. In another study, a transcriptomic survey of stress response-associated genes revealed that exposure of tomato plants to arsenic up-regulated glutathione reductase (LeGR). This inhibitory effect was mitigated by the addition of Si in the form of CaSiO_3_ [142]. In 2015, Zhu et al. [93] suggested that Si can improve salt tolerance of cucumber plants through up-regulation of the central plasma membrane aquaporin gene expression. Whereas, in the case of pea, recently in 2017, Rahman et al. [143] provided the mechanistic evidence on the beneficial effect of Si on Cd toxicity in pea plants, and transcriptome analysis revealed a predominant up-regulated expression of GSH1 (phytochelatin precursor) and MTA (metallothionein) transcripts in roots and down-regulated expression of pea Fe transporter (RIT1) in shoots.

In the case of proteomics, Chen et al. [74] explored the role of Si-mediated resistance to *Ralstonia solanacearum* in tomato root by a proteomics approach. They identified a total of 53 proteins. Forty-eight out of 53 proteins were significantly influenced by Si application. On these lines, a proteomics study in *Capsicum annuum* revealed that Si treatment up-regulated the accumulation of proteins involved in several metabolic processes, particularly those associated with transferase activity and nucleotide binding and modulated the expression of proteins involved in ubiquitin-mediated nucleosome pathway and carbohydrate metabolism [79]. In 2017, Bityutski et al. [109] highlighted the importance of both Fe and Si supply in plant exclusion of Al under acidic conditions; they reported that Si modulated the increase in root succinate and facilitated the long-distance transport of Fe, thereby hindering Al transport from roots to shoots. Recently, Ali et al. [144] reported that Si is vital in regulating the metabolic content of tomato leaves under osmotic stress. They observed a change in the metabolite profile in roots (22) and leaves (27), respectively.

## 6. Silicon Transporters

Si is absorbed by the plant roots in the form of silicic acid [145]. High Si accumulation in plants has been attributed to an efficient Si uptake system. However, to our knowledge, the molecular mechanism for Si uptake in vegetable crops is less understood and has only been reported in pumpkin [146,147], soybean [148], and cucumber [55,149]. For the first time in the dicotyledonous crop, an influx transporter of Si (*CmLsi1*: *CmLsi1* B+ and *CmLsi1* B^−^) was identified in two pumpkin cultivars, significantly differing for Si accumulation. Si transporters, expressing in all root cells were localized plasma membrane and other at the endoplasmic reticulum (ER) [146]. Si uptake by plants is controlled by the actions of influx (Lsi1) and efflux (Lsi2) transporters. Deshmukh et al. [148] identified, characterized, and cloned two putative Si transporter genes, *GmNIP2-1* and *GmNIP2- 2* from soybean. Both genes, localized at the plasma membrane were expressed in shoot and root tissues. Two putative Si transporter genes (*CSiT-1* and *CSiT-2*) have also been cloned and characterized in cucumber plants [131]. Recently, Sun et al. [149] isolated and characterized a gene *CsLsi1,* encoding a Si influx transporter in cucumber that shared around 55.70% and 90.63% homology with the Lsi1s of rice and pumpkin, respectively. This gene was localized at the plasma membrane for expression in roots. 

## 7. Conclusions

Si is among the abundant elements on the earth. Here, we have discussed the role of Si application in protecting vegetable crops against several biotic and abiotic stresses. Although, Si absorption and availability to vegetables under natural conditions is low. The forms of Si commonly used for the plant application are potassium silicate, silica sol, slow-and NH4-silicates, etc. With the advancement in the omics-based approaches, information regarding the role of Si, in shaping vegetable crops protection against abiotic and biotic stress is increasing. Further research regarding the uptake of Si by vegetables, to determine the newly available form of Si for plants, as well as the mechanisms behind Si application and plant stress elevation, has to be determined. This review focused on the role of silicon application for vegetables. For cereals, Si is on the verge of becoming a regular fertilizer, and we hope for an imitative trend in vegetables. The pathway of silicon, even in extensively cultivated vegetables remains to be explored. Hence, there is a need to employ omics-based approaches to identify in details the pathways and the genes responsible for the Si uptake by the vegetables to develop vegetable varieties with better Si uptake mechanisms.

## Figures and Tables

**Table 1:** Summary of the effects of Si application against biotic stresses.

Vegetable Crop	Form of Silicon Applied	Observed Effect of Silicon	Reference
**Fungal Pathogen**			
*C. sativus*	Silicate fertilizer	Promoted the growth and yield and also reduced the damage caused by wilt disease	[60]
*Cucumis sativus*	Soluble silicates	Reduced the size of fungal colonies (*S. fuliginea*)	[27]
*C. sativus*	Soluble silicon	Decreased the receptivity of plants to mildew infection caused by *S. fuliginea*	[61]
*C. sativus*. and *Solanum lycopersicon*	Potassium silicate	Reduced the infection caused by *Pythium* and *F.solani*	[28]
*C. sativus*	Potassium silicate added to hydroponic nutrient solutions	Suppressed powdery mildew (PM) caused by *S.fuliginea*	[62]
*C. sativus*	Soluble silicon	Significantly decreased the powdery mildew disease (caused by *S. fuliginea*) index	[29]
*C. sativus*	Potassium metasilicate	Significantly suppressed powdery mildew (*P. xanthi*)	[14]
*C. melo*	Sodium silicate and nanosized silicon	Significantly decreased the severity of mildew powder	[35]
*S. lycopersicon* and *M. charantia*	Silicic acid	Symplastic Si was associated with the reduction of the spread of the fungus (*P. aphanidermatum*) in roots	[31]
*C. sativus*		Significantly reduced the incidence of damping-off (*P. aphanidermatum*)	[32]
*C. melo*	Sodium silicate	Reduced the postharvest rot (*T. roseum*)	[39]
*C. sativus*		Significantly decreased the disease index (*S. fuliginea*)	[63]
*C. melo*	Potassium silicate	Reduced the severity and incidence of powdery mildew (*S. fuliginea*)	[36]
*C. pepo*	Potassium silicate	Enhanced the tolerance to salinity and resistance to powdery mildew (*P. xanthii*)	[40]
*C. sativus*	Sodium silicate	Reduced downy mildew (*P. cubensis*) disease index	[33]
*C. annuum*	Calcium silicate	Potentially reduce the severity of Phytophthora blight	[45]
*C. sativus*	Sodium silicate	Enhanced crop resistance to oxidative stress induced by *P. melonis* infection	[30]
*S. lycopersicon*	Sodium metasilicate nonahydrate	Reduced the disease severity of Fusarium crown and root rot (*F. oxysporum* f.sp. radicis-lycopersici)	[43]
*G. max*	Wollastonite	Controlled the soybean rust (*P. pachyrhizi*)	[49]
*C. melo*	Potassium silicate	Controlled the powdery mildew (*P. xanthi*)	[37]
*C. sativus*	Carbon Silpower solution	Inhibited powdery mildew (*P. xanthi*) development	[64]
*G. max*	Potassium silicate	Protected plants against soybean rust (*P. pachyrhizi)*	[48]
*S. lycopersicon*	Sodium silicate	Suppressed anthracnose disease (*C. gloeosporiodes*)	[44]
*S. lycopersicon*	Potassium silicate	Reduced the severity and incidence of Fusarium wilt (*F. oxysporum* f. sp. lycopersici)	[65]
*S. lycopersicon*	Potassium silicate	Reduced Fusarium wilt (*F. oxysporum* f. sp. lycopersici)	[66]
*C. annuum*	Potassium silicate	Enhanced resistance to anthracnose (*Colletotrichum gloeosporioides*)	[46]
*S. lycopersicon*	Sodium silicate	Controlled anthracanose disaese (*C. gloeosporioides*)and improved postharvest quality of fruits	[67]
*M. charantia*	Potassium silicate	Strengthened resistance in plants against powdery mildew (*Erysiphe sp.*)	[38]
*C. pepo*	Calcium silicate, CaMg silicate slag, wollastonite and MontanaGrowTM	Suppressed Powdery mildew (*P. xanthii*)	[41]
*S. lycopersicon*	Silicon rich rice hull	Enhanced anthracnose resistance (*C. dematium*)	[68]
*C. sativus*	Sodium silicate	Enhanced resistance to Fusarium wilt (*F. oxysporum* f. sp. cucumerinum Owen) and altered soil microbial communities	[34]
**Bacterial pathogen**			
*S. lycopersicon*	Monosilicic acid	Acted as an inducer of resistance against *R. solanacearum*	[50]
*S. lycopersicon*		Significantly reduced the incidence of bacterial wilt (*R. solanacearum*)	[51]
*S. lycopersicon*	Soluble silicon	Reduced wilt incidence ( *R. solanacearum*)	[53]
*S. lycopersicon*	Monosilicic acid and aerosol powder	Induced basal resistance against *R. solanacearum*	[42]
*S. lycopersicon*		Reduced severity and incidence of bacterial wilt (*R. solanacearum*)	[69]
*S. lycopersicon*	Monosilicic acid	Induced resistance against *R. solanacearum*	[52]
*S. lycopersicon*	Monosilicilic acid	Induced resistance against bacterial wilt (*R. solanacearum*)	[70]
*S. lycopersicon*	Supa Sílica and calcium silicate	Reduced the symptoms of bacterial speck ( *P. syringae* pv. Tomato)	[71]
*S. lycopersicon*	Potassium silicate	Controlled *R. solanacearum* incidence by changing the soil microorganism amount and enzyme activity	[54]
*Cucumis melo* L.	Calcium silicate	Induced resistance against bacterial fruit blotch *(A. citrulli*)	[72]
*S. lycopersicon*	Monosilicic acid	Induced systemic resistance against bacterial wilt (*R. solanacearum*)	[73]
*S. lycopersicon*		Suppressed bacterial wilt (*R. solanacearum*)	[55]
*S. lycopersicon*	Potassium silicate	Induced resistance against bacterial wilt (*R. solanacearum*)	[74]
*Cucumis melo* L.	Calcium silicate	Enhanced resistance to bacterial fruit blotch (*A. citrulli*)	[75]
**Insect pest and nematodes**			
*C. sativus*	Calcium silicate	Acted as resistance Inducers against the Whitefly (*B. tabaci*)	[19]
*G. max*	Silicic acid	significantly decreased the Silverleaf whitefly populations	[56]
*S. lycopersicon*	AgrosilícioTM	Controlled leafminer (*T. absoluta*) owing to toxic and anti-feeding effect to the larval stage	[58]
*C. sativus*	Potassium silicate	Acted as an anti-herbivore defense	[57]
*C. sativus*	sodium metasilicate	Significantly reduced the activity of root-knot nematode (*M. incognita*)	[59]

**Table 2:** Summary of the effects of Si application against abiotic stresses.

Vegetable Crop	Form of Silicon Applied	Observed Effect of Silicon	Reference
**Salinity**			
*C. sativus*	Potassium silicate	Alleviated salt stress and increased antioxidant enzymes activity	[83]
*S. lycopersicon*	Sodium silicate	Alleviated salt toxicity	[122]
*S. lycopersicon*	Potassium silicate	Alleviated the deleterious salt effect	[89]
*C. sativus*	Potassium silicate	Alleviated the salinity stress	[123,124]
*Spinacia oleracea*	Sodium silicate	Increased stress tolerance	[84]
*C. sativus*		Enhanced salinity tolerance	[125]
*M. charantia*	Potassium silicate	Alleviated salt stress and increases antioxidant enzymes activity	[85]
*G. max*	Sodium metasilicate	Alleviated the detrimental effect of salinity stress	[126]
*C. sativus*	Sodium silicate	Increased resistance against salinity	[127]
*Trigonella foenumgraceum*	Sodium silicate	Increased the tolerance to salt stress	[128]
*S. lycopersicon*		Alleviated the effect of salinity stress	[129]
*S. lycopersicon*	Silicon and nano silicon	Improved the salt tolerance	[82,130]
*C. sativus*	Sodium silicate	Alleviated salt-oxidative stress	[86,93]
*Cucurbita pepo*	nano-SiO2	Improved the defense mechanisms of plants against salt stress toxicity	[92]
*C. sativus*	Silicic acid	Enhanced the salt tolerance	[131]
*Pisum sativum*	Potassium silicate	Alleviated the salinity-induced deleterious effects	[90]
*C. annumn*	Nano Silicon	Improved salinity tolerance	[91]
*S. lycopersicon*	Sodium silicate	Alleviated salinity stress	[132]
*C. sativus*	Sodium silicate	Alleviated oxidative damage and improved plant growth and photosynthetic performance	[95,133]
*C. annuum*	Potassium silicate	Mitigated salinity stress	[94]
*S. lycopersicon*	Nano-silicon	Regulated the expression of salt tolerance genes under salinity stress	[134]
*S. tuberosum*	Silicon dioxide nanoparticles	Improved the salinity tolerance	[135]
*S. lycopersicon*	Potassium silicate	Manipulated ion Distribution of plants under salt stress	[136]
*S. lycopersicon*	Silicic acid	Improved nutrient levels and yields under saline conditions	[137]
*S. lycopersicon*	Silicon nanoparticles	Enhanced salinity tolerance and improved plant growth with exopolysaccharide-producing bacteria	[138]
**Drought**			
*Capsicum annuum*		Increased the tolerance to water deficit	[96]
*C. annuum*	Sodium metasilicate	Alleviated negative effects of water deficiency	[97]
*G. max*	Sodium metasilicate	Alleviated seedling damage under drought and ultraviolet-B radiation	[88]
*G. max*	Silicic acid	Mitigated the adverse effects of salt and drought stress	[98]
*S. lycopersicon*	Sodium metasilicate	Increased total chlorophylls under water-deficient conditions	[139]
*S. lycopersicon*	Silicic acid	Improved seed germination and alleviated oxidative stress under water deficit stress	[99]
*S. lycopersicon*	Sodium silicate	Restrained chlorophyll degradation and increased optimal photosynthetic efficiency under drought stress	[100,101,103]
*S. lycopersicon*	Potassium silicate	Enhanced the water stress tolerance	[102,140]
**Mineral toxicity**			
*G. max*	Soluble silicon	Alleviated the symptoms of Al toxicity	[108]
*Vigna unguiculata*	Potassium silicate	Alleviated Mn toxicity	[111]
*C. sativus*	Potassium silicate	Alleviated the Mn toxicity	[112]
*C. sativus*	Silicic acid	Alleviated Mn toxicity and modulated the metabolism and utilization of phenolic compounds	[113]
*C. sativus*	Sodium silicate	Alleviated the adverse effects of excess Mn and cadmium (Cd) toxicity	[114,118]
*C. sativus*		Improved antioxidant capacity of plant under Cd toxicity	[141]
*C. sativus*	Silicic acid	Ameliorated manganese toxicity by decreasing hydroxyl radical accumulation	[115]
*S. lycopersicon*	Calcium silicate	Mitigated the inhibitory effects of arsenic	[142]
*S. lycopersicon* and *C. sativus*	Sodium metasilicate nonahydrate	Alleviated Cd stress	[119]
*C. sativus*	Sodium silicate	Alleviated autotoxicity caused by 3-phenyl propionic acid during seed germination	[87]
*C. sativus*	Silicic acid	Enhanced leaf remobilization of iron under limited iron conditions	[116]
*C. sativus*	Potassium silicate	Mitigated the toxicity of ammonium	[120]
*S. lycopersicon*	Monosilicic acid	Mitigated ammonium toxicity	[121]
*C. sativus*	Sodium silicate	Alleviated autotoxicity and Cd toxicity	[87]
*C. sativus*	Silicic acid	Mitigated the Al toxicity under acidic conditions	[109]
*P. sativum*	Orthosilicic acid	Alleviated Cd toxicity	[143]
*S. tuberosum*	Sodium silicate	Improved the defense ability against Al toxicity	[110]
**Osmotic**			
*C. sativus*		Induced alleviation of growth reduction under osmotic stress	[104]
*C. sativus*	Silicon spray	Enhanced the capacity of scavenging active oxygen species and improved photosynthesis	[105]
*C. sativus*	Sodium metasilicate	Contributed tolerance against osmotic stress	[106]
*S. lycopersicon*	Monosilicic acid	Regulated osmotic stress tolerance by differential accumulation of relevant amino acids	[144]
**Cold**			
*C. sativus*	Potassium silicate	Provided chilling tolerance	[107]
**UV-B**			
*G. max*	Sodium metasilicate	Enhanced nutrient acquisition under UV-B Radiation	[22,88]
